# A Survey of Bacterial Microcompartment Distribution in the Human Microbiome

**DOI:** 10.3389/fmicb.2021.669024

**Published:** 2021-05-13

**Authors:** Kunica Asija, Markus Sutter, Cheryl A. Kerfeld

**Affiliations:** ^1^Environmental Genomics and Systems Biology Division, Molecular Biophysics and Integrated Bioimaging Division, Lawrence Berkeley National Laboratory, Berkeley, CA, United States; ^2^Michigan State University-U.S. Department of Energy (MSU-DOE) Plant Research Laboratory, Department of Biochemistry and Molecular Biology, Michigan State University, East Lansing, MI, United States

**Keywords:** bacterial microcompartments, metabolosomes, pathogenesis, human microbiome, dysbiosis

## Abstract

Bacterial microcompartments (BMCs) are protein-based organelles that expand the metabolic potential of many bacteria by sequestering segments of enzymatic pathways in a selectively permeable protein shell. Sixty-eight different types/subtypes of BMCs have been bioinformatically identified based on the encapsulated enzymes and shell proteins encoded in genomic loci. BMCs are found across bacterial phyla. The organisms that contain them, rather than strictly correlating with specific lineages, tend to reflect the metabolic landscape of the environmental niches they occupy. From our recent comprehensive bioinformatic survey of BMCs found in genome sequence data, we find many in members of the human microbiome. Here we survey the distribution of BMCs in the different biotopes of the human body. Given their amenability to be horizontally transferred and bioengineered they hold promise as metabolic modules that could be used to probiotically alter microbiomes or treat dysbiosis.

## Introduction

**FIGURE 1:**
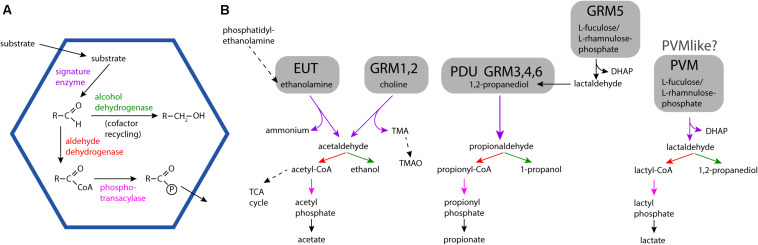
General BMC reaction overview and detailed reaction pathways for different BMC types found in microbiome samples. **(A)** General encapsulated pathway scheme of metabolosomes. **(B)** Substrates, intermediates and products of the EUT, PDU, GRM, and PVM/PVM-like BMCs. Solid arrows indicate BMC-associated reactions. TMA: trimethylamine; TMAO: trimethylamine-N-oxide; TCA cycle, tricarboxylic acid cycle; DHAP, dihydroxyacetone phosphate.

Bacterial Microcompartments (BMCs) are organelles that are functionally similar to those of eukaryotes; they establish and contain a microenvironment that is distinct from the rest of the cell (Kerfeld et al., 2018; Kirst and Kerfeld, 2019). BMCs are bounded by a selectively permeable membrane, however, in contrast to their eukaryotic counterparts, this membrane, —the shell— is composed of proteins. All BMC shells are assembled from homologous building blocks, enabling their bioinformatic identification in genomic sequence data (Axen et al., 2014; Bobik et al., 2015; Zarzycki et al., 2017; Sutter et al., 2021). BMCs are either anabolic, such as the extensively studied carboxysome (Kerfeld and Melnicki, 2016) or catabolic; these are collectively known as metabolosomes (Figure 1A). An aldehyde intermediate is common to the encapsulated chemistry of many metabolosomes and the enzyme generating the aldehyde is referred to as the signature enzyme (Axen et al., 2014; Kerfeld and Erbilgin, 2015). The purpose of the BMC shell in these metabolosomes is to enhance catalysis and sequester toxic aldehyde intermediates (Figure 1B) (Kerfeld et al., 2018). Gut bacteria often have the potential to form ethanolamine utilization (EUT) BMCs because ethanolamine is abundant in the intestine as a breakdown product of phosphatidylethanolamine (Larson et al., 1983). Indeed, the EUT operon is part of the core *E. coli* genome (Dadswell et al., 2019), allowing the organism to use ethanolamine as a source of both carbon and nitrogen (Kaval and Garsin, 2018). Because it is an environment with a large spectrum of available substrates known to be catabolized within BMCs, they are frequently found in gut microbes (Ravcheev et al., 2019).

Encompassing the gut and beyond, the human microbiome has been defined as an essential organ of the human body given the tremendous effects it has on overall health (Shreiner et al., 2015; Kashyap et al., 2017; Manor et al., 2020; Fan and Pedersen, 2021). Dysbiosis, or disruption of a healthy microbiome, has been implicated in obesity, hypertension, cardiovascular disease, diabetes, cancer and even depression (Ding et al., 2019). Although little is known about the exact mechanism with which the microbiome exerts its influence, it seems plausible that the BMCs could potentially play a role in these outcomes by enabling the dominance of specific taxa. Although yet to be experimentally verified, BMC expression likely exerts some influence on the community composition by conferring the ability to catabolize niche-specific metabolites. Here we survey the available census of human microbiome organisms to identify their BMCs. Our compilation provides the first insights into the prevalence of specific types BMCs in particular niches, and suggests associations with BMC-based metabolism and the nutritional landscape of the sampling site in health and disease.

## Methods

Tables matching up bacterial strains with body locations were downloaded from the Pathosystems Resource Integration Center (PATRIC)^1^, the Human Oral Microbiome Database (HOMD)^2^, and the NIH Human Microbiome Project^3^. Strain names were the matched with the assigned loci described in (Sutter et al., 2021) and correlated with body sites found in the respective databases (Supplementary Table 1). Duplicates with identical NCBI taxid and body site were removed.

## Results

### EUT BMCs Are Commonly Found to Be Associated With the Gut and Oral Environments

**FIGURE 2:**
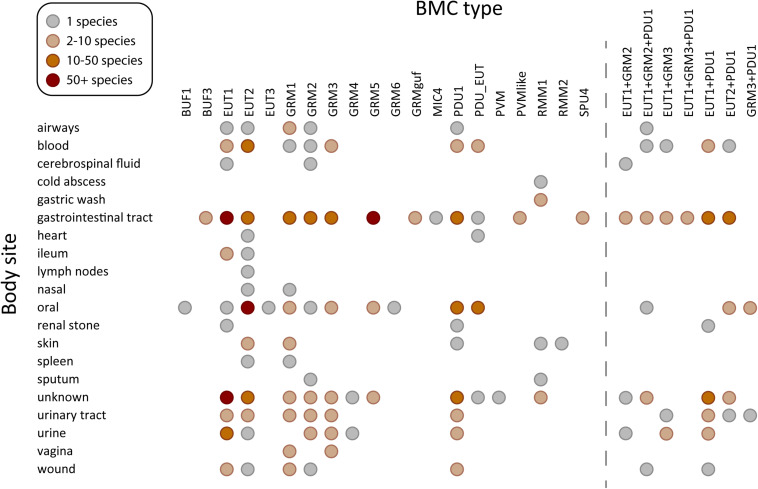
Species containing BMC functional types found in human microbiome sampling sites. BMC types found in sequenced genomes of 625 species in the human body plotted against the site or source from which they were isolated. Acronyms for the BMC functional types (Axen et al., 2014; Sutter et al., 2021): PDU, propane diol utilization; GRM, glycyl radical enzyme containing microcompartment; GRMguf, GRM with unknown function glycyl radical enzyme; EUT, ethanolamine utilization microcompartment; BUF, bacterial microcompartment of unknown function; RMM, *Rhodococcus* and *Mycobacterium* microcompartment; PVM/PVM-like, *Planctomycete* and *Verrucomicrobia* microcompartment; SPU, sugar phosphate utilizing microcompartment; PDU_EUT, genetic fusion of the PDU/EUT loci; MIC, microcompartments with an incomplete core. BMC types co-occurring in the same species, observed in at least in three species, are shown on the right.

The EUT BMCs allow organisms to utilize ethanolamine as a carbon and nitrogen source by metabolizing it into acetaldehyde and ammonia using the encapsulated ethanolamine ammonia lyase (Tsoy et al., 2009). There are three major types of EUT BMCs, namely EUT1, EUT2, and EUT3. Loci of these three types all encode the signature enzyme ethanolamine ammonia lyase but differ in the genes encoding ancillary proteins, regulatory proteins as well as the type and presence of core metabolosome elements (Sutter et al., 2021). In humans, ethanolamine is obtained through the diet as the product of the breakdown of the common lipid component of plant and animal cell membranes, phosphatidylethanolamine or from the breakdown of phospholipids in normal turnover of epithelial cells. Accordingly, organisms containing EUT BMCs are enriched in the gastrointestinal tract (Figure 2), or other sites with epithelial turnover. Ethanolamine is abundant in the inflamed gut and the presence of tetrathionate as the electron acceptor allows for intestinal pathogens such as *Salmonella enterica*, *Enterococcus faecalis*, enterohaemorrhagic *Escherichia coli* (EHEC), *Clostridium difficile* to flourish by utilizing EUT BMCs (Bertin et al., 2011; Srikumar and Fuchs, 2011; Thiennimitr et al., 2011; Anderson et al., 2018; Ormsby et al., 2019). Likewise, the ability to derive carbon, nitrogen and energy from ethanolamine is a hallmark of urinary tract infections, in which *E. coli* inhabit successively the perineum, the urethra and the bladder. The EUT BMC is directly involved in this progression (Sintsova et al., 2018; Dadswell et al., 2019).

BMCs are also found in organisms in cancer-associated gut dysbiosis. *Fusobacterium hwasooki* and *F. nucleatum* (Supplementary Table 1) species contain a EUT2 BMC, and *F. nucleatum* specifically is suggested to play a role in the progression of colorectal cancer (CRC) as well as oral squamous cell carcinoma (Zhou et al., 2018; Zhang et al., 2019). The microbial community in the oral microenvironment and its imbalance has likewise been implicated in diseases such as periodontitis and in dental caries (Lamont et al., 2018). There are studies showing direct correlation between EUT and periodontitis progression (Kaval and Garsin, 2018). All three major EUT BMC types are found in organisms populating the oral microbiome, underscoring the importance of ethanolamine degradation in this environment (Figure 2). The EUT2 BMC type was the most commonly found EUT subtype present in the genera *Leptotrichia*, *Streptococcus* and *Fusobacterium* (Supplementary Table 1) which are all members of the oral microbiome (Deo and Deshmukh, 2019). Furthermore, it has been shown that in the presence of ethanolamine, there was an increase in the respiratory activity of several of the pathogenic organisms in the oral microbiome (Hernandez-Sanabria et al., 2017) indicating that this is an important metabolite, and the ability to form a EUT BMC would provide a competitive advantage.

### The Distribution of the PDU1 BMC Ranges From the Respiratory System to the Gut

PDU (1,2-propanediol utilization) BMCs have a wide distribution comparable to EUT BMCs and they commonly co-occur in many different organisms (Sutter et al., 2021; Figure 2). In some species, such as strains of *Streptococcus* and *Listeria monocytogenes*, the two loci are fused (PDU_EUT, Supplementary Table 1). In other organisms, like *F. nucleatum*, the EUT and PDU BMCs are likely differentially regulated by the locus-encoded regulator, depending on availability of substrates. Via the diet, plant sugars rhamnose and fucose are catabolized by organisms in anaerobic conditions to produce 1,2-propanediol (1,2-PD), which can be utilized by PDU BMCs as a source of carbon and energy (Figure 1B). *Lactobacillus panis* and *Velionella denticariosi*, which can form PDU BMCs, were identified in the oral microbiome (Supplementary Table 1) and both are associated with human dental caries (Byun et al., 2004, 2007). Within the gut, *Shigella flexneri*, *Citrobacter* sp., and *Lactobacillus brevis* (Supplementary Table 1) all encode PDU1 BMCs in their genomes. Both *Citrobacter* and *Lactobacillus* species are implicated in human gut dysbiosis in patients suffering from irritable bowel syndrome (Ganji et al., 2016). Organisms containing PDU BMCs are also found in the blood and vagina (Figure 2). The presence of 1,2-propanediol in the vagina could be an indicator of a healthy microbiome; a study in 2013 showed that women with bacterial vaginosis had a reduction in the presence of 1,2-propanediol which is formed by the hydrogenation of lactic acid (Yeoman et al., 2013). *Lactobacillus*, which is a hallmark organism of a healthy vaginal microbiome, was not identified in this survey. *F. nucleatum*, identified in the vagina harboring the EUT1 and PDU1 BMCs (Supplementary Table 1) has been associated with bacterial vaginosis. This organism has also been implicated in causing preterm birth as well as intrauterine infections (Agarwal et al., 2020).

### GRM Microcompartments Are Widespread in the Human Microbiome

GRM1 (glycyl radical enzyme containing microcompartment) and GRM2 BMCs produce TMA as a side product of choline metabolism (Figure 1B). TMA can be absorbed and oxidized to trimethylamine-N-oxide (TMAO) by flavin-containing monooxygenases (Fennema et al., 2016). TMAO is known to contribute to cardiovascular disease (Wang et al., 2011; Schiattarella et al., 2017). TMAO is also generated by the gut microbiome after consumption of foods rich in L-carnitine and phosphatidylcholine, such as fish, eggs and red meat (Zeisel et al., 1983; Wang et al., 2011). The abundance of GRM1 and GRM2 BMCs in the gut organisms (Figure 2) reflects that the gastrointestinal tract is rich in choline. Some of the organisms in the gut that contain the GRM2 BMCs include the known pathogen *Providencia alcalifaciens*, the causative agent of foodborne illnesses (Shah et al., 2019) and *Klebsiella* sp. which is an opportunistic pathogen (Supplementary Table 1). Additionally, GRM2 was identified in various strains of *E. coli* (Supplementary Table 1). The presence of the GRM BMCs in the blood and urine could be accounted for by sepsis along with another cardiovascular related health conditions that result in the formation of TMAO. Indeed, GRM1 and GRM2 can be found in the majority of bacterial species associated with urinary tract infections. Recently, a GRM2 BMC, encoded in a pathogenicity island, has been shown to be involved in choline utilization in *E. coli* UPEC 536 (Herring et al., 2018).

GRM3, GRM4 and GRM6 BMCs are functionally analogous to PDU BMCs (Levin and Balskus, 2018; Ferlez et al., 2019) and consistently show a similar microbiome distribution as species containing PDU BMCs (Figure 2). The GRM5 BMCs have additional enzymes that enable it to process fuculose/rhamnulose phosphate which are typical degradation products of complex polysaccharides. This is consistent with the occurrence of many GRM5-containing species in the gastrointestinal microbiome (Figure 2). One of the GRM5 containing organisms in gut is the anaerobe *Ruminococcus gnavus* (Supplementary Table 1) that is known to be associated with Crohn’s disease (Henke et al., 2019).

### Recently Discovered, Less Characterized BMCs Are Found in Members of the Human Microbiome

Several newly discovered or less familiar microcompartments such as RMM and the PVM-like have been identified in our human microbiome survey (Figure 2). The RMM organelles are named for representatives found in *Rhodococcus* and *Mycobacterium* species (Axen et al., 2014). This BMC has been proposed to metabolize aminoacetone to propionyl-CoA (Mallette and Kimber, 2018). Organisms containing RMM1 were identified in the skin microbiome (*Rhodococcus erythropolis* SK121) and the gut (various strains of Mycobacteria) (Supplementary Table 1). There have been studies showing the presence of *Mycobacterium* species with cutaneous infections and conditions such as cold abscess (Franco-Paredes et al., 2018; Bains et al., 2019). It is likely that the ability to utilize aminoacetone could provide a competitive advantage in the disease state. Interestingly, none of the common pathogenic mycobacterial strains such as *M. tuberculosis* and *M. leprae* contain the RMM1 BMC. There is a single occurrence of RMM2 in *Paracoccus yeei* TT13 that was found in a skin sample (Supplementary Table 1) and shown to grow on 1,2-PD as sole carbon and energy source (Lim et al., 2018).

PVM microcompartments encapsulate a class-II aldolase as their signature enzyme with the substrates rhamnose and fucose (Erbilgin et al., 2014; Figure 1B). They are primarily found in environmental samples where they are thought to metabolize algal cell wall degradation products (Sichert et al., 2020; Sizikov et al., 2020). The PVM-like BMC locus contains a claas-II aldolase homolog aldolase and an aldehyde dehydrogenase that is expected to process 1,2-PD (Sutter et al., 2021), so a similar substrate as PVM is likely. Species containing the PVM-like microcompartments can be identified in gastrointestinal tract where they likely also play a role in the breakdown of complex carbohydrates (Figure 2); these organisms include *Hungatella hathewayi*, *Clostridium bolteae*, and *Clostridium* sp. AF18-27 (Supplementary Table 1). One of the PVM-like BMC containing organisms is *Faecalibacterium prausnitzii*, which has been shown to be beneficial for gut health in a mouse model system (Munukka et al., 2017).

The sugar phosphate utilizing microcompartments (SPU) are emerging as one of the most widespread types of BMCs; they are predicted to be involved in DNA catabolism via the deoxyribose/deoxyribulose 5-phosphate degradation pathway (Axen et al., 2014; Sutter et al., 2021). The degradation of exogenous DNA, a common component of detritus, is a source of carbon and energy (Finkel and Kolter, 2001). Organisms containing SPU4 BMCs such as *Anaerotruncus colihominis*, *Clostridium* sp. AF15-17LB and *Dorea* sp. D27 have been found in the gastrointestinal tract samples (Supplementary Table 1), consistent with availability of nucleic acid from the turnover of resident microorganisms.

### Microcompartments of Unknown Functions Are Found in the Gut and Oral Microbiome Organisms

BUF microcompartments or Bacterial Microcompartments of Unknown Function loci encode the structural proteins to form the metabolosome shell but not an aldehyde dehydrogenase (Axen et al., 2014). A BUF1 has recently been characterized as a compartment for the potential degradation of xanthine (encapsulating Xanthine dehydrogenase) (Ravcheev et al., 2019). Elevated levels of metabolites including xanthine, hypoxanthine, inosine have been detected in the metabolome of periodontitis and gingivitis associated oral samples (Duran-Pinedo and Frias-Lopez, 2015) and a single BUF1 BMC containing organism (*Bacillus* sp. 2_A_57_CT2, Supplementary Table 1) has been found in the oral microbiome (Figure 2).

Not much is known about the Microcompartments with Incomplete Core (MIC), except that they contain an aldehyde dehydrogenase (Sutter et al., 2021) and a class II aldolase that may imply a similar function as the PVM BMCs. A single organism (*Lachnospiraceae bacterium* KGMB03038) containing the MIC4 BMC has been identified in the stool sample of a healthy person (Supplementary Table 1).

## Discussion

With the increasing availability of bacterial genome sequences, including those from culture independent genomic methods and microbiomes, the number and diversity of known BMCs is rapidly increasing (Sutter et al., 2021). In many ecosystems the BMCs employed by community members reflect important characteristics of the nutritional landscape of the environmental niche, such as the importance of the PVM BMC (Planctomycete and Verrucomicrobia microcompartment) for the degradation of complex polysaccharides originating from algae (Erbilgin et al., 2014; Sichert et al., 2020; Sizikov et al., 2020). In the human microbiome, pathogenic bacteria are able to gain a fitness advantage by catabolizing organic compounds that are metabolically unavailable to the native microflora (Passalacqua et al., 2016). For example, numerous studies show the role of the PDU BMC in the proliferation and persistence of pathogens. This compound is naturally present in the gut as a by-product of microbial fermentation of the sugars rhamnose and fucose (Badia et al., 1985; Schardt et al., 2017). During colonization, effector molecules cause inflammation of the intestine subsequently forming tetrathionate (Chowdhury et al., 2014). Tetrathionate is utilized as the electron acceptor by EUT-containing organisms, conferring a distinct competitive advantage, in conjunction with 1,2-propanediol, also found in the gut (Thiennimitr et al., 2011). This allows opportunistic gut pathogens such as *Salmonella* to survive in anaerobic conditions by not only using tetrathionate as the terminal electron acceptor but also by cobalamin synthesis, which requires anaerobiosis, the expression of which is co-regulated with genes from the PDU locus (Jakobson and Tullman-Ercek, 2016). The PDU gene cluster has been implicated in providing *L. monocytogenes* with a significant fitness advantage in the gastrointestinal tract as evidenced by faster clearing of infection in murine models infected with *pdu* deletion mutants (Schardt et al., 2017; Zeng et al., 2019). It is becoming increasingly apparent that many organisms have the potential to form more than one functional type of BMC (Figure 2) (Sutter et al., 2021); such as *Salmonella enterica* which contains both PDU and EUT BMCs (Stojiljkovic et al., 1995), and organisms associated with urinary tract infections (Sutter et al., 2021).

The types of BMCs in organisms of the human microbiome sampling sites reflect the local nutritional landscape; for example, the EUT, PDU, and GRM BMCs are gut associated. Similarly, the oral microbiome has a predominance of the GRM and EUT BMC types. The GRMs constitute three functionally distinct types based on the substrate of the GRE (Zarzycki et al., 2015; Figure 1B). GRM1 and GRM2 are associated with anaerobic breakdown of choline to trimethylamine (TMA) and acetaldehyde intermediates (Figure 1B). Interestingly, the conversion of choline to TMA is only possible through microbial activity (Craciun and Balskus, 2012; Craciun et al., 2014). The GRM3/4/6 organelles use the GRE 1,2-PD dehydratase to convert 1,2-PD into propanol and propionate (Zarzycki et al., 2017; Schindel et al., 2019) and GRM5 is involved in the anaerobic degradation of rhamnose/fuculose (Petit et al., 2013; Zarzycki et al., 2015). Collectively, we find these BMCs are the most prevalent in available sequence data from human microbiome samples.

In addition, several newly discovered BMCs such as RMM, PVM and PVM-like have been discovered to be associated with human microbiome albeit some of them from undefined sites of the human body. Uncharacterized BMCs such as BUF and MIC were identified in the gut and stool samples. The experimental characterization of these metabolic modules is complementary to metabolomics studies of these microenvironments because the function of the BMCs reflects the metabolic profile of the microenvironments.

The metabolic profile of sampling sites is regulated by several factors; diet, the propensity for host cell breakdown (epithelial layers) and the composition of the microbiome are primary determinants (Valdes et al., 2018; Leeming et al., 2019; Visconti et al., 2019; De Angelis et al., 2020). One study finds increased expression of the EUT genes in *Listeria monocytogenes*, anaerobically and in the presence of vitamin B12 (Zeng et al., 2020). While this does not provide a direct correlation between change of diet in humans and its effect on BMC gene expression, it does demonstrate that these genes are induced by available substrates.

The catabolic activity of BMCs within the human microbiome likely impacts the metabolic profile of a particular site. Given that the future of personalized medicine likely includes routine site-specific sampling of a patient’s microbiome throughout life, monitoring organism composition, and its metabolic potential may emerge as a means to manage homeostasis and health. Likewise, BMC-based manipulation of the microbiome could offer an approach to treating dysbiosis. A pathogen can colonize a given environment if it has the ability to use a limiting or specific nutrient (Freter et al., 1983). BMCs endow organisms the ability to catabolize substrates metabolically unavailable to commensals for a competitive advantage. In a microbiome-based approach, endowing a probiotic organism with a BMCs used by a pathogen may provide a way to outcompete it.

According to the World Health Organization, as of 2019 communicable diseases such as lower respiratory and diarrheal are among the top 10 causes of death globally. Bacteria responsible for causing lower respiratory illnesses include members of the *Streptococcus* genera, *E. coli, Klebsiella pneumoniae, Mycoplasma pneumoniae*, and *Mycobacterium tuberculosis* (Dasaraju and Liu, 1996). Similarly, urinary tract infections are the primary source for outpatient infections in the United States and are caused by organisms including uropathogenic *E. coli* (UPEC), *K. pneumoniae*, and *Pseudomonas aeruginosa*, Group B *Strep* (Medina and Castillo-Pino, 2019). Many of the organisms implicated in causing these diseases encode BMCs in their genomes.

The microbiome can be thought of as a pliable ecosystem that can be altered to have immense overall impacts on human health. Altering the microbiome using an individual-based approach to account for the variation may aid in resolving complex issues such as obesity and inflammatory bowel disease (Wang et al., 2020). This can further be improvised by the addition of engineered probiotic strains. BMCs, as metabolic modules encoded by genetic modules, provide a way to introduce by “plug and play” expanded metabolic potential into probiotic organisms. Engineering BMCs for use in microbiome-based therapies can be thought of as an additional approach in the field of precision medicine. BMCs encapsulate necessary enzymes for substrate utilization and can be engineered to include enzymes that will breakdown a disease-causing substrate or even potentially enclosing a toxic intermediate. Indeed, developing engineered BMCs that have a high bacterial host range and are easy to modulate (Kirst and Kerfeld, 2019) may be achievable in the foreseeable future.

## Author Contributions

CAK conceived the project and wrote the manuscript. KA and MS generated and analyzed the dataset and wrote the manuscript. All authors read and approved the manuscript.

## Conflict of Interest

The authors declare that the research was conducted in the absence of any commercial or financial relationships that could be construed as a potential conflict of interest.

## Footnotes

1 www.patricbrc.org

2 www.homd.org

3 www.hmpdacc.org
